# Sociodemographic predictors of latent class membership of problematic and disordered gamblers

**DOI:** 10.1016/j.abrep.2016.04.004

**Published:** 2016-04-16

**Authors:** Richard J.E. James, Claire O'Malley, Richard J. Tunney

**Affiliations:** a School of Psychology, University of Nottingham, University Park, Nottingham NG7 2RD, UK; b School of Psychology, University of Nottingham Malaysia Campus, Semenyih, Selangor, Kuala Lumpur, Malaysia

**Keywords:** Disordered gambling, Gambling, Addictive behaviours, Impulsivity

## Abstract

This paper reports a series of analyses examining the predictors of gambling subtypes identified from a latent class analysis of problem gambling assessment data, pooled from four health and gambling surveys conducted in Britain between 2007 and 2012. Previous analyses have indicated that gambling assessments have a consistent three class structure showing quantitative and potentially qualitative differences. Bringing this data together is useful for studying more severe problem gamblers, where the small number of respondents has been a chronic limitation of gambling prevalence research. Predictors were drawn from sociodemographic indicators and engagement with other legal addictive behaviours, namely smoking and alcohol consumption. The pooled data was entered into a multinomial logistic regression model in which class membership was regressed along a series of demographic variables and survey year, based on previous analyses of gambling prevalence data. The results identified multiple demographic differences (age, general health, SES, being single, membership of ethnic minority groups) between the non-problem and two classes endorsing some problem gambling indicators. Although these two groups tended to share a sociodemographic profile, the odds of being male, British Asian and a smoker increased between the three groups in line with problem gambling severity. Being widowed was also found to be associated with the most severe gambling class. A number of associations were also observed with other addictive behaviours. However these should be taken as indicative as these were limited subsamples of a single dataset. These findings identify specific groups in which gambling problems are more prevalent, and highlight the importance of the interaction between acute and determinant aspects of impulsivity, suggesting that a more complex account of impulsivity should be considered than is currently present in the gambling literature.

## Highlights

• Data was pooled from three health and gambling surveys to identify correlates of gambling subtypes.
• Subclinical gambling problems correlated with being male, single, a smoker, and under 35.
• Problematic gambling was more common in multiple ethnic minority, and some socioeconomic groups.
• The most severe gambling group had greater odds of being male, British Asian, a smoker and widowed.

## 1 Introduction

The aims of population-wide measurements of disordered gambling are to examine or uncover trends in gambling involvement and assess whether problem gambling prevalence is changing. Identifying these trends is crucial to directing appropriate resources towards reducing or mitigating harm and informing interventions, particularly as disordered gambling appears to show considerable heterogeneity and may require distinct treatment goals (Blaszczynski & Nower, 2002). There is also a close degree of correspondence between the assessments used in UK gambling prevalence research and screens administered by healthcare practitioners to gamblers seeking treatment (Bowden-Jones & George, 2015). Recent commentaries have suggested that rather than comparing disordered gambling prevalence across timeframes or jurisdictions, the greatest benefit from prevalence research has emerged from comparing across sub-samples of gambler (Markham & Young, 2016). This paper pools data from multiple British surveys using similar survey designs to uncover the predictors of latent class membership from socio-demographic correlates and other addictive behaviours, building on latent class analyses (LCAs) of problem gambling assessments that have consistently observed three subtypes of gambler (McBride et al., 2010, Carragher and McWilliams, 2011, James et al., 2016). Pooling data has the potential to be beneficial in uncovering the demographic correlates of those showing the greatest difficulties with gambling, where individual gambling surveys have tended to be unable to sample enough of these gamblers to draw strong inferences about this group.

Previous LCAs of disordered gambling data have indicated that the measures of pathological gambling included in representative samples of the British population have a similar latent structure that appears to be similar across time. LCAs have been conducted on two adaptations of the DSM-IV Pathological Gambling criteria (American Psychiatric Association, 2000), the South Oaks Gambling Screen (Lesieur & Blume, 1987) and the Problem Gambling Severity Index (Ferris & Wynne, 2001), suggesting that a broadly similar profile emerges (James et al., 2016). These tended to produce consistent results which suggest the presence of three interpretative categories of gambler across the measurements analysed. These identify an initial category of gamblers who have minimal likelihood of endorsing a problem gambling indicator, making up 85–95% of the sample, a second category of gamblers who showed some problems with gambling but mostly at a sub-clinical level (with endorsement primarily limited to loss-chasing and preoccupation indicators) and a third category of gamblers all of whom exceeded the most severe category of the instrument being used. These categories appeared to be quantitatively and qualitatively distinguishable. Subtypes differed in problem severity and showed relatively little overlap, strongly indicative of a dimension of severity. However, the indicators that showed maximal differences between the second and third highest severity categories were the loss of control items, similar to other analyses of problem gambling data testing the presence of a latent category (James, O'Malley, & Tunney, 2014).

The British Gambling Prevalence Survey (BGPS) was a series of nationally representative surveys that assessed gambling attitudes and behaviours, and problem gambling prevalence, between 1999 and 2010 in the United Kingdom (Sproston et al., 2000, Wardle et al., 2007, Wardle et al., 2011). The first survey was conducted in light of major changes to the gambling market over the 1990s, and the second and third were conducted to provide baseline and follow-up measurements in light of major gambling legislation (the Gambling Act 2005, enacted in July 2007). Further data was also collected in a module of the Health Survey for England 2012 and the Scottish Health Survey 2012. The survey in 2010 (Wardle et al., 2011) found a significant increase in the prevalence of ‘problem’ gambling between 2007 and 2010, using an assessment that was adapted from the DSM-IV Pathological Gambling criteria (*p* = .046). Although the DSM criteria doesn't have a subtype of problem gambling, a cutoff of three has often been used to identify individuals who exhibit significant subclinical difficulties with gambling (Sproston et al., 2000, Chou and Afifi, 2011, Nower et al., 2013). This increase was identified using a logistic regression model in which problem gambling status was predicted for each survey year, age, sex, marital status, ethnicity, socio-economic status, general health status and incidence of cigarette smoking. Many caveats were applied to this finding at the time, as the authors of the BGPS report noted that other, unobserved factors may explain this difference (Wardle et al., 2011). Recent commentaries (Sharman, Aitken, & Clark, 2014) have pointed out that the absolute number of individuals driving this difference was very small; for example, the 2010 dataset contained around twenty additional problem gamblers, with both surveys having fewer than one hundred problem gamblers each. This highlights one of the limitations of using gambling prevalence survey data to compare between subgroups of gambler (Doughney, 2007, Lorains et al., 2011). Although it is desirable to make comparisons across data that can generalised to the wider population it has proven to be highly problematic because of the difficulties in sampling a sufficient number of the gamblers reporting the greatest number of problems to uncover consistent associations. Pooling data across surveys can potentially make this problem more tractable. The British prevalence data lends itself better than many other datasets to pooling because the different studies had similar approaches to sampling and weighting, recruited similar sample sizes and used the same problem gambling assessments that have a similar latent class structure. The response rates across the surveys are similar (52%, 47%, 56%), and are much higher than some other gambling prevalence surveys (Markham & Young, 2016), where responses have fallen as low as 20%. The British prevalence surveys also appear to concord with many of the best practices identified by Williams and Volberg (2010).

Nevertheless, there are a number of caveats that result from pooling data from the datasets covered in this analysis, in addition to the limitations associated with gambling prevalence surveys. To start, the amount of missing data for problem gambling assessments is different between the surveys conducted. The completion rates across the three datasets amongst the respondents who were administered them were 89.97% (BGPS 2007), 99.75% (BGPS 2010) and 88.94% (SHS & HSE 2012). The higher completion rate on the BGPS 2010 data is likely due in part to the utilisation of a computer aided procedure to administer the questionnaire, whereas the other surveys were paper based. In addition, only around three in four respondents (77.39%) to the HSE/SHS surveys were asked any questions from the gambling module. It is unclear whether the difference between the respondents who were given the gambling module or not was random or systematic. The BGPS and HSE/SHS surveys were framed very differently to one another; the British Gambling Prevalence Survey was presented as a leisure survey, but the problem gambling questions were situated towards the end of an extensive questionnaire probing gambling behaviour. The Health Survey for England was explicitly framed as a health questionnaire, and asked a range of questions about health and wellbeing related behaviours. The way in which a gambling questionnaire is framed has an important impact on estimates of gambling involvement (Williams, Volberg, & Stevens, 2012), with health surveys eliciting lower rates of responding to questions about gambling behaviour.

Although there are important limitations with comparing across the different sets of data, we believe that the potential benefits outweigh the costs. As mentioned previously the greater sample of problem gamblers allows identification of commonalities, if any exist, where it has been difficult to do so previously. The health survey data contains more granular data on a number of areas pertinent to gambling, particularly on other licit addictive behaviours such as drinking and smoking. Given that models of problem gambling identify the role of impulsive personality traits and hypothesize that the causal mechanism behind the most severe problem gamblers is a common risk factor for addictive behaviours, comparing across sub-samples using this data can provide broader information on the interaction between gambling and addictive behaviours across a wider spectrum. Some of this data has been utilised previously. Wardle et al. (2014) used alcohol and smoking frequency data from two health surveys in studying the predictors of at risk gambling (defined as a score between 3 and 7 on the PGSI), and problem gamblers (identified using either the PGSI or DSM screen), using a logistic regression procedure to compare between these groups and respondents who did not fall into the target group (or a higher severity group). This was based on a simulated stepwise procedure to determine which predictors were significant from a set of socio-economic and health indicators. These other addictive behaviours, along with being more likely to be younger, male and Muslim, were associated with ‘at risk’ gambling, but not problem gambling. The health survey data includes a wider range of data about these behaviours that may provide valuable insights into the engagement gamblers have with other addictive behaviours, including several variables not considered in previous analyses. There is also the issue that coding the DSM data using the underlying logic of the DSM (i.e. a behaviour is classified as present or absent) identifies a much greater rate of endorsement than the PGSI, with around twice as many gamblers typically endorsing a problem gambling behaviour than using the PGSI (McBride et al., 2010, James et al., 2016). This also applies to the proportions of at-risk and problematic gamblers.

In this report we examine the correlated of subtypes of problem gambling derived from latent class modelling. A three latent class model was estimated as previous research that has found this consistently captures the different subtypes of gambler that emerge from gambling assessment data. From this, we estimated a multinomial logistic regression using the most likely latent class each case belonged to as the outcome variable. We subsequently examined the relationship between gambling and smoking and alcohol use in the health survey data.

## 2 Method

### 2.1 Sample

This study pooled data from past-year gamblers that completed the problem gambling assessment derived from the DSM-IV Pathological Gambling criteria in the BGPS 2007 (*n* = 5503), BGPS 2010 (*n* = 5699), and combined data from the SHS 2012 and HSE 2012 (*n* = 6909), resulting in a total sample of 18,111 respondents. Latent class analysis was conducted using MPlus version 6.1.1 (Muthén & Muthén, 1998-2011). The other analyses were conducted in STATA v. 14 SE (StataCorp, 2015). The data was collected by the National Centre for Social Research in 2007, 2010 and 2012, and is publicly available from the UK Data Archive (National Centre for Social Research, 2008, National Centre for Social Research, 2011, National Centre for Social Research, and University College L, Scottish Centre for Social Research, University College Lon, Scottish Centre for Social Research et al., 2015).

The statistical analyses were adjusted for survey design. The datasets include probability weights that can be used to adjust the samples to the ONS mid-point population estimates for the year the data was collected in. Further variables are included in the dataset to adjust for the primary sampling unit respondents were drawn from and stratification. For the multinomial logistic regression analysis two strata had to be merged into the subsequent stratum because there would have only been one primary sampling unit in the strata with non-missing data on at least one of the variables. Weighted demographic data are reported in Table 1.

**Table 1:** Weighted count data for the sociodemographic indicators.

Variable	Class 1 (*n* = 16,716)	Class 2 (*n* = 1281)	Class 3 (*n* = 267)
*Sex:*			
Male	7994 (7780, 8209)	887 (809, 965)	215 (180, 251)
Female	8274 (8068, 8481)	394 (349, 438)	52 (37, 67)

*Age:*			
18–24	1853 (1718, 1987)	349 (297, 400)	67 (46, 88)
25–34	2681 (2540, 2822)	339 (291, 387)	71 (51, 90)
35–44	3166 (3014, 3318)	238 (204, 273)	56 (39, 73)
45–54	2946 (2809, 3083)	173 (144, 202)	44 (29, 58)
55–64	2633 (2508, 2758)	99 (78, 121)	18 (10, 26)
65–74	1752 (1662, 1841)	58 (43, 72)	10 (4, 16)
75 +	1232 (1149, 1314)	25 (15, 36)	2 (− 1. 4)

*Smoking status:*			
Yes	3866 (4305, 4662)	490 (438, 543)	127 (101, 154)
No	13,232 (12,905, 13,559)	784 (715, 853)	140 (112, 167)

*Marital status:*			
Married/civil partnership	10,220 (9932, 10,508)	607 (551, 663)	106 (84, 129)
Separated or divorced	1376 (1296, 1455)	95 (74, 117)	25 (14, 36)
Single	3564 (3387, 3741)	544 (479, 609)	126 (98, 153)
Widowed	964 (899, 1029)	25 (16, 35)	8 (3, 14)

*Ethnicity:*			
White British	15,338 (14,982, 15,694)	1126 (1040, 1211)	206 (173, 238)
Mixed	166 (132, 200)	24 (13, 35)	8 (2, 15)
Asian British	354 (294, 414)	58 (36, 79)	27 (15, 40)
Black British	270 (225, 316)	46 (29, 63)	17 (6, 28)
Chinese British/other	87 (63, 111)	22 (11, 32)	8 (0, 15)

*Socio-economic status:*			
Professional/managerial	6535 (6293, 6778)	410 (357, 462)	70 (49, 91)
Intermediate occupation	1597 (1486, 1709)	150 (120, 180)	16 (8, 25)
Small employer/self-employed	1765 (1634, 1896)	128 (102, 154)	27 (14, 39)
Lower supervisory/technical	1745 (1624, 1866)	122 (96, 147)	29 (13, 44)
Semi-routine occupation	4112 (3930, 4295)	393 (345, 441)	105 (83, 127)

Note: There are missing data in a number of these instances.

## 3 Analytic procedure

### 3.1 Latent class analysis

A weighted LCA was conducted on individual items from the DSM-IV Pathological Gambling criteria, coded as present/absent in the manner as other LCAs of British Pathological Gambling data (McBride et al., 2010, James et al., 2016). Only a three class model was estimated as it appears that this is consistent across multiple surveys. LCA is a method of identifying distinct subtypes within a latent categorical variable. It assumes that both the manifest and latent variables in the analysis are categorical, and that the indicators entered into the analysis are independent from one another at the level of the latent class. This assumption of local independence was tested by examining the Chi-square test of overall model fit in the output, which indicated that the assumption was met (*p* > 0.05).

## 4 Regression analysis

Sociodemographic indicators were entered into a logistic regression model with most likely latent class as the outcome variable, adopting an identical approach where possible to the analysis conducted by Wardle et al. (2011). Covariates were selected on the analysis conducted by Wardle et al. (2011). The variables included were as follows:

– Survey year (2007, 2010 and 2012).
– Ethnicity (categorised as White British/non-British, mixed ethnic background, Asian British, Black British and Chinese British or other ethnicity).
– Socio-economic status (NS-SEC 5 category classification used — managerial/professional occupation, intermediate occupation, small employers and own-account workers/self-employed, lower supervisory and technical occupations, and semi-routine occupations).
– Marital status (married/living as married/civil partnership, separated/divorced, single (never married), and widowed).
– Self-reported health status (measured on a five point scale from ‘very good’ to ‘very bad’, with ‘fair’ as the middle option).
– Present smoking status (yes/no).
– Age (categorised into seven bands, < 24, 25–34, 35–44, 45–54, 55–64, 65–74, 75 +).
– Sex (male/female).

All variables apart from self-reported health were dummy coded. The reference categories for each variable are reported in Table 2. The ethnicity variables for the BGPS 2007 and SHS/HSE datasets were recoded to cover the same categories as the BGPS 2010 data, because the number of categories differed between surveys. For the HSE & SHS 2012 data, this meant referring back to the original SHS & HSE data files downloaded from the UK Data Agency (National Centre for Social Research, and University College L, Scottish Centre for Social Research, University College Lon). The ID variable (an eight digit number) and age data were used to match respondents, and the ‘origin’ variable, which asks about ethnicity in greater detail, was used to generate commensurate groups. Some categories of the marital status variable were merged for the same reason.

**Table 2:** Multinomial logistic regression of demographic variables on latent class membership. The intermediate severity class (class 2) is the reference class.

Variable — class 1 v class 2	RRR	Std. error	*t*	*p*	95% CI
*Year (ref: 2007)*					
2010**	0.775	0.066	− 3.000	0.003	0.655, 0.916
2012	1.191	0.118	1.770	0.076	0.982, 1.446

*Ethnicity (ref: White British/non-British)*					
Mixed	0.742	0.218	− 1.010	0.312	0.417, 1.322
British Asian**	0.542	0.131	− 2.530	0.011	0.337, 0.871
Black British***	0.439	0.091	− 3.950	< .001	0.292, 0.661
British Chinese/other ethnicity***	0.300	0.088	− 4.090	< .001	0.169, 0.535

*Socio-economic status (ref: professional/managerial)*					
Intermediate occupation**	0.662	0.082	− 3.310	0.001	0.519, 0.846
Small employer or self-employed	0.894	0.114	− 0.880	0.379	0.696, 1.148
Lower supervisory or technical occupation	0.958	0.119	− 0.340	0.731	0.75, 1.224
Semi-routine occupation**	0.738	0.072	− 3.100	0.002	0.609, 0.895

*Marital status (ref: married)*					
Separated/divorced	0.797	0.105	− 1.720	0.086	0.614, 1.033
Single**	0.760	0.074	− 2.800	0.005	0.628, 0.921
Widowed	1.123	0.286	0.460	0.648	0.682, 1.852

*Age (ref: <=24)*					
25–34	1.194	0.151	1.400	0.162	0.931, 1.531
35–44***	2.005	0.266	5.240	< .001	1.545, 2.602
45–54***	2.655	0.368	7.040	< .001	2.022, 3.485
55–64***	4.198	0.739	8.150	< .001	2.971, 5.93
65–74***	4.640	0.823	8.650	< .001	3.276, 6.573
>= 75***	6.938	1.954	6.880	< .001	3.992, 12.058
Smoker (ref: yes)***	1.466	0.114	4.940	< .001	1.259, 1.707
General health***	0.783	0.035	− 5.410	< .001	0.717, 0.856
Sex (ref: female)***	2.409	0.184	11.530	< .001	2.074, 2.798


Variable — class 3 v class 2	RRR	Std. error	*t*	*p*	95% CI
Year (ref: 2007)					
2010	1.092	0.206	0.470	0.638	0.755, 1.58
2012	0.918	0.204	− 0.380	0.701	0.594, 1.419

*Ethnicity (ref: White British/non-British)*					
Mixed	1.331	0.679	0.560	0.576	0.489, 3.621
British Asian*	2.286	0.815	2.320	0.021	1.136, 4.601
Black British	1.673	0.636	1.350	0.176	0.793, 3.526
British Chinese/other ethnicity	1.948	1.348	0.960	0.335	0.501, 7.575

*Socio-economic status (ref: professional/managerial)*					
Intermediate occupation	0.622	0.208	− 1.420	0.156	0.323, 1.199
Small employer or self-employed	1.070	0.351	0.200	0.838	0.562, 2.037
Lower supervisory or technical occupation	1.247	0.429	0.640	0.520	0.635, 2.449
Semi-routine occupation	1.395	0.305	1.520	0.129	0.908, 2.143

*Marital status (ref: married)*					
Separated/divorced	1.353	0.382	1.070	0.284	0.778, 2.353
Single	1.352	0.297	1.370	0.170	0.879, 2.081
Widowed**	3.788	1.701	2.970	0.003	1.57, 9.14

*Age (ref: <=24)*					
25–34	1.101	0.303	0.350	0.726	0.642, 1.89
35–44	1.394	0.417	1.110	0.268	0.775, 2.509
45–54	1.369	0.484	0.890	0.374	0.684, 2.741
55–64	0.944	0.390	− 0.140	0.889	0.42, 2.123
65–74	0.704	0.316	− 0.780	0.434	0.291, 1.699
> = 75	0.246	0.209	− 1.650	0.098	0.047, 1.298
Smoker (ref: yes)*	0.701	0.115	− 2.160	0.031	0.507, 0.968
General health	1.204	0.115	1.950	0.052	0.999, 1.451
Sex (ref: female)**	0.541	0.107	− 3.120	0.002	0.368, 0.797

* = < .05.

** = < .01.

*** = < .001.

There are multiple approaches that have been used to explore the effect of covariates on latent class membership. Feingold, Tiberio, and Capaldi (2014) outline one, three and revised three step approaches. The three step approach, in which most likely latent class membership is included as a predictor in a logistic regression with covariates as the indicator variables, was used in this case. In some cases it has been demonstrated that this approach is inappropriate because it underestimates the effect size and standard errors for the potential covariates. In contrast, with a one-step approach the covariates and latent regression are included in the latent class model. However, this is equally troublesome; Vermunt (2010) critiques this approach because it requires re-estimation of the latent class model every time a covariate is added or removed, and adds additional computing time to conducting an analysis, as well as going against the intuitive logic of building a statistical model. Instead, a modified three-step approach was proposed in which the third step (regressing most likely class membership on covariates) is modified using a maximum likelihood correction that was subsequently found in simulations to produce more accurate estimates when the classes are well separated. However, studies of real world and simulated data have indicated when the entropy (a measure of classification accuracy) of a latent class model is greater than 0.8, then a traditional three-step logistic regression procedure including most likely latent class membership as the predictor variable produces accurate estimates that do not excessively inflate or deflate standard errors (Clark & Muthén, 2009). The entropy of the latent class model was 0.895, meaning that a three step approach was appropriate for this data. The intermediate severity group was chosen as the reference class to examine differences between intermediate and high severity gamblers.

## 5 Results

### 5.1 Latent class analysis

The estimated three class model identified one class that showed only a small probability of endorsing any of the pathological gambling indicators, a second class that had a high probability of endorsing the preoccupation and between-session loss-chasing indicators and a low probability of the remaining indicators and a third class that had a high to moderate probability of endorsing most indicators, but showed the largest differences on loss of control related items (pathological gambling indicators 3–7). The indicators strongly differed quantitatively, with relatively little overlap on overall symptom count; the first class endorsed either zero or one of the DSM criteria, the second class between one and five indicators (the majority between one and four) and the third class more than five. The third class typically endorsed five or more criteria, on average endorsing between six or seven. Indicators three through seven showed similar probabilities of responding (between 0.755 and 0.84). These show relatively large differences in relative rates of endorsement but quite small in absolute terms (all are endorsed by between 2 and 4% of the sample), and item response theory analyses of these data suggest that these span the dimension of severity that has been observed using the DSM data (Strong & Kahler, 2007).

### 5.2 Covariate analysis

Table 3 provides the full results of the logistic regression model (Table 2 reports count data for each variable in the regression). A number of differences were observed between the group showing minimal or no problems, and the group endorsing some problem gambling indicators. Gamblers in the intermediate group were around twice as likely to report coming from a Black or Asian British background, and three times more likely to come from another ethnic minority, relative to a White British background. They were one and a half times more likely to be a smoker, and two and a half times more likely to be male.

**Table 3:** Smoking and alcohol indicators across the different latent classes in the combined Health Survey for England and Scottish Health Survey 2010 datasets.

Variable	Class 1 (*n* = 6241)	Class 2 (*n* = 396)	Class 3 (*n* = 93)	Linear regression/Chi-square test
Current smoking status:				χ (6) = 2.95, *p* = 0.009
Never	2939	166	47	
Ex-occasional smoker	343	22	3	
Ex-regular smoker	1635	81	15	
Regular smoker	1323	127	28	
Number of cigarettes smoked:				
Weekday	11.897	12.961	13.613	N.S.
Weekend	13.158	13.482	15.716	N.S.
Smoking frequency (ex-smokers):				χ (4) = 19.71, *p* = 0.07
Regularly	1635	343	155	
Occasionally	81	22	7	
Only tried once or twice	15	3	7	
Advised by doctor to quit smoking				χ (2) = 2.38, *p* = 0.09
Yes	842	62	19	
No	2404	158	24	
Number of units:				Class 2 > Class 1
Drank per week	12.545	16.396	16.694	
Unit risk status:				χ (4) = 5.19, *p* = < .001
Low	4369	1650	244	
Increasing	235	131	32	
Higher	67	18	8	

Between the low and intermediate severity groups, there were also a number of significant differences amongst the sociodemographic correlates that subsequently did not differ between the intermediate and higher severity groups. These included socioeconomic grouping, marital status, self-reported general health and membership of an ethnic minority. General health did not differ between the intermediate and higher severity groups (*p* = 0.052, 95% CI = 0.999–1.45). Relative to the moderate severity latent class, on four indicators there were greater log odds of being found in the third or most severe latent class: whether the respondent was a current smoker, male, British Asian or widowed. Three of these were also significant between the lowest severity class and the reference group, suggesting that these track alongside problem gambling severity. Although the three classes differed in overall severity (i.e. problem gambling score), many differences one might expect between the latent classes, such as perceived general health and age (disordered gambling is more prevalent in younger individuals) failed to emerge.

### 5.3 Smoking

One finding of particular interest was that smoking prevalence tracked alongside problem gambling severity. Theoretical models of problem gambling claim that the most severe problem gamblers are characterised by antisocial and impulsive personality traits, and that these gamblers should show a common risk of addictive behaviours. From the Health Survey data it is possible to get more detailed information about prevalence of smoking, amount of cigarettes smoked per day and previous engagement with smoking, whereas the gambling data only includes current smoking status. In the HSE 2012 dataset there were 1560 current smokers (22.57% of the sample). Table 3 reports the descriptive statistics concerning smoking. Of particular interest was that it appeared that fewer individuals in the most severe gambling group had never smoked relative to the other two classes, as well as are more likely to be current smokers; the two more severe gambling groups trended towards having a lower prevalence of social/occasional smokers than the least severe gamblers. Across all groups present smokers tended to smoke one to two additional cigarettes on a typical weekend day relative to a weekday. This has previously been identified in studies of university students (Colder et al., 2006). However, there was no evidence that the number of cigarettes smoked was associated with class membership. Amongst ex-smokers, the pattern of smoking behaviour was relatively constant across groups; around 3 in 4 ex-smokers reported being regular smokers, with the remainder of occasional and rare (i.e. 1 or 2 cigarettes) being evenly distributed. There were a couple of potential areas where trends were observed that could not be conclusively established due to the low number of respondents (only around half of the most problematic gamblers, already a very small group, smoked). The survey data also queried whether respondents had been advised by their medical practitioner to quit smoking. As with smoking frequency, there was a trend with class membership, but this was not significant. This might be of interest for further research.

### 5.4 Alcohol use

To look at alcohol consumption, we regressed the number of average units drank per week on latent class membership, with the recreational gambler group used at the reference category. This revealed that the second group (showing preoccupation and loss-chasing behaviours) consumed a significantly greater number of units than the recreational group (*b* = 4.60, SE = 1.61, *p* = .004, 95% CI = 1.443–7.766) but that the most severe gamblers did not (*b* = 3.031, SE = 3.81, *p* = .43, 95% CI = − 4.453, 10.514). In addition, there was a significant association between alcohol risk group and gambling latent class. Using the Chief Medical Officer's Guidelines of < 14 units (both genders), as ‘low risk’, 14–49 units as ‘increasing risk’, and 50 + units as ‘higher risk’, there was a significant association between latent class and risk group (Table 3).

## 6 Discussion

The results of these analyses identify a number of sociodemographic characteristics that predict membership of latent classes derived from indicators of disordered gambling. Compared to the reference class (who tended to endorse the loss-chasing and preoccupation indicators), the subgroup endorsing minimal to zero gambling problems were less likely to come from semi-routine and intermediate occupational groups, less likely to come from a number of ethnic minority groups (Black British and Chinese British/other ethnicity), reported better general health, less incidence of smoking and was more likely to be female. The most severe problem gamblers were more likely to be male, a current smoker, come from a British Asian background and divorced. Latent class membership also appeared to be associated with multiple different types of engagement with drinking and smoking.

The analysis compared differences between latent classes on a number of demographic attributes. In some instances, the proportion of members belonging to a certain group or engaging in a specific behaviour tracked alongside latent class membership and thus severity. The odds of being male or a smoker increased with membership of a higher problem gambling severity latent class. The likelihood of a class member being British Asian also increased with latent class severity, with 1.7%, 3.9% and 8.75% of the low, moderate and high severity classes coming from this group. The other ethnic minority groups (mixed ethnicity individuals aside) were more likely to be in the intermediate class relative to the low gambling severity class, but there were no differences in membership between the second and third severity classes (although all had greater odds of being in the problem group too). It has been frequently observed that men have higher prevalence of numerous addictive disorders (Keyes et al., 2010, Khan et al., 2013), although women show a ‘telescoping’ effect in which initiation of drug, drinking or gambling begins later but the transition to disordered behaviour is shortened (Keyes et al., 2010, Grant et al., 2012). Studies of younger cohorts suggest that these differences might be diminishing (Keyes et al., 2010), but caution should be applied in comparing between timeframes, as critiques of prevalence studies have pointed out that structural changes in responding mean that this might be at least partially artefactual (Markham & Young, 2016). The demographic differences between the second and third latent classes were relatively minor. As noted above, the odds of the second and third classes differing on most demographic variables were small. Combined with the findings from previous LCAs of this data, this should be taken as stronger evidence that the primary difference between these groups lies in a loss of control over gambling but with the caveat that more intensive research with a subgroup of these gamblers would be highly informative.

The prevalence of problem gambling is higher in more disadvantaged socioeconomic groups, although with some assessments this relationship has been confounded by a preponderance to assess disordered gambling using items related to excessive monetary spending or borrowing. Research that has looked at the density of gaming machines, which are typically associated with harmful play, has found that these are more common in more deprived areas (Wardle, Keily, Astbury, & Reith, 2012). This study found that respondents from this group were less likely to show very few gambling problems, in line with most of the literature on this topic (Welte, Barnes, Wieczorek, Tidwell, & Parker, 2002). In addition, respondents from ‘intermediate occupations’, a more affluent group, were also less likely to be in the group with the least gambling problems. This is potentially a group requiring further study. Similar findings have been found in alcohol, where pockets of greater consumption have been identified amongst comparatively better off drinkers (Jones, Bates, McCoy & Bellis, 2015). Further scoping research would be beneficial to study gambling behaviours amongst this group.

Previous research has found an elevated risk of problem gambling amongst British Asian adolescents (Forrest & McHale, 2012). Using pooled adult and adolescent data (including the BGPS 2007/2010 data), a similar finding was observed, with a significantly higher level of problem gambling amongst British Asian women (Forrest & Wardle, 2011). While we broadly replicate this finding, as the ratio of British Asian to White British problem gamblers was 8.34 (versus 1.99 for males), this should be taken with extreme caution as only six female British Asian problem gamblers were identified across the three weighted samples. As noted by Forrest and Wardle (2011), the BGPS 2007 identified that British Asians held some of the most negative attitudes towards gambling, although this may be a consequence of the increased prevalence of problem gambling amongst this community. A similar finding was observed in widowed people, another group where attitudes towards gambling were similarly negative (Wardle et al., 2007). There is a broader evidence base here, as a literature has developed looking at gambling in older people. The Pathways Model predicts that traumatic life events are associated with certain pathways to problem gambling, and previous analyses based on directly testing this model included recent family death as an indicator in a LCA of US data, finding that more severe pathological gamblers tended to have a higher probability of reporting a recent bereavement (Nower et al., 2013).

A number of socio-demographic variables appear to map onto constructs related to risk-taking and impulsivity, which are known to be associated with increased endorsement of disordered gambling indicators. The issue of the relationship between gambling and smoking has been investigated (Petry & Oncken, 2002), but is somewhat less well explored than associations between gambling and other drug addictions (McGrath & Barrett, 2009). This research indicates that many of these gamblers smoke, but potentially are more likely to be advised by a clinician to quit.. It should be noted that the age distribution of the two classes endorsing problem gambling items is similar to that of smokers in the UK (HSCIC, 2015). This also has a wider impact as there is a preponderance towards focusing on the individual nature of disordered gambling behaviour, in contrast to the addiction literature, which has recognised the influence of acute exposure on state impulsive behaviour (de Wit, 2009), including acute nicotine exposure (Hogarth, Stillwell, & Tunney, 2013). The high levels of cigarette use observed in the most problematic gamblers highlight that many gamblers will be involved with numerous behaviours that appear to increase the likelihood of engaging in further risk taking behaviours such as gambling, or certain types of gambling behaviour. The problem gambling literature has extensively studied the trait or determinant aspects of impulsivity, repeatedly finding that problem and pathological gamblers show higher self-reported and behavioural levels of trait impulsivity, particularly when the questionnaire content probes retrospective behaviour (Fortune & Goodie, 2010). However, the issue of state impulsivity has been relatively sparsely addressed in this context. There are two potential benefits in doing so. The first is that it is well established, and further found here, that the most severe levels of problem gambling are comorbid with other addictive behaviours. Studying these acute effects has the potential to further our understanding of the relationship between gambling, addictive behaviours and impulsivity, as it may be the case that these state effects are associated with certain features or sequences of risky gambling activity. It may also be the case that gambling exhibits a similar effect on other addictive behaviours. The second is that in the wider addiction literature, gambling has the potential to be the most interesting probe of this problem as the acute effects of gambling can be (ethically) manipulated by altering the schedule of reinforcement, whereas the opportunity for doing so in with substance use is more constrained. The gambling literature has noted the presence of dissociative experiences, stereotypically in machine gambling play. Further research on this matter has the potential to contribute to an important issue in the addiction literature, where it has been argued that the study of gambling has had less of an impact than might be expected (Cassidy, 2014).

Our findings have a potential impact in the context of public health and campaigns designed to raise awareness of problem gambling. We identified similar demographic profiles in the two groups that systematically endorsed problem gambling indicators. It is common to target specific populations in the information materials and interventions aimed at public health priorities. The data brought together in this study allows clearer identification of which groups problem gambling is more likely to be found. This approach is already being taken in some instances, with recent campaigns by industry self-regulatory bodies that have been specifically aimed at younger men. These analyses identify groups where a targeted focus might be beneficial with a view towards designing messaging that is relevant to them — one of the issues with problem gambling (and addictive behaviours in general) is the low levels or treatment seeking amongst those experiencing the greatest harm or with a use disorder. In understanding the demographic correlates of different gambling groups it is possible to direct further research towards identifying the products or behaviours that may be the target of intervention in the future. Concerns have been raised that the choice of location of gambling products has been the source of consistent criticism from gambling pressure groups. It has often been claimed that gambling locations are set up in communities where problematic gambling is more common, and gambling pressure groups have recently accused some operators of targeting the placement of shops in areas with majority ethnic minority populations (Ramesh, 2016), who in this study were consistently associated with endorsing problem gambling behaviours.

There are some limitations with this analysis relating to the datasets used. The first is that the sampling or administration method changed between surveys, even if the questionnaire content was identical. For instance, the BGPS 2010 introduced a computer aided self-interview schedule. More substantially, a considerable minority of respondents to the HSE & SHS datasets were not administered the gambling module. It is unclear whether the respondents who were and were not administered the questionnaire significantly differed in any sense. In addition the use of a past-year gambling criterion for administering problem gambling assessments has been identified as potentially problematic due to the risk of false positives, primarily when very low problem gambling thresholds are used (Williams et al., 2012). While it is frequently noted that one of the advantages of using a nationally representative survey is to draw conclusions about the wider population, research on the correspondence between prevalence surveys and clinical assessment has been modest at best. However, this again tends to occur when lower thresholds are used. Moreover, because many of the demographic variables (ethnicity in particular, but also SES and marital status to an extent) have the vast majority of respondents affirming one or two categories, the confidence intervals are quite broad, particularly for comparisons against the most severe gamblers. This is also the case for the smoking and drinking data, where the subsamples of the three classes were analysed. While a number of effects are not significant, the truncated sample means that it would be premature to claim that there is no effect in a number of these cases. However, these are indicative of where more intensive research may be beneficial on specific subsamples of gambler.

Although the latent classes identified have been consistently found using different samples and across different jurisdictions, the use of gambling prevalence survey data is ultimately limited in this regard. LCA does not provide a conclusive answer concerning the qualitative differences between gambling subtypes, particularly as the subtypes are accompanied by a notable difference in symptom count. Analyses that tend to be more sensitive in identifying qualitative differences (McGrath & Walters, 2012) have suggested that some of the most severe problem gamblers form a taxon (Kincaid et al., 2013, James et al., 2014). However while these analyses show greater sensitivity, these only inform the presence of a qualitative difference and not the number of distinct subtypes. Self-reported gambling assessments are also likely to under and over represent responding in certain contexts (Doughney, 2007). The LCA findings, taken with other latent variable analyses are indicative of a mixed latent structure. However, given the restricted set of indicators and small samples of problem gambler, more in-depth research perhaps with a sample of highly engaged gamblers might begin to tease out some of the differences that emerge in these groups in further detail. These analyses provide broad indications concerning where these differences may lie, but research going beyond gambling prevalence would need to be conducted to directly test this.

To conclude, sociodemographic predictors of latent classes derived from gambling assessment data were studied on pooled data from four surveys. Overall there appeared to be more similarities than differences between moderate and severe problem gamblers. Gamblers showing some problems but not meeting the clinical threshold for Pathological Gambling tended to be male, single, younger, come from a number of ethnic minority backgrounds, smoke, report poorer general health and emerge from two specific socioeconomic strata (intermediate and semi-routine occupations). The most severe gamblers, relative to the group showing problems, were more likely still to be male, smoke and come from a British Asian background. There was also an association between membership of the most severe gambling group and being widowed. These predictors appeared to be stable across the different datasets.

## Conflict of interest

The authors declare that there are no conflicts of interest.

## Funding statement

This work was supported by the Economic and Social Research Council (ES/J500100/1) and the Engineering and Physical Sciences Research Council (EP/G037574/1).
